# Improving the time-efficiency of initial mental health assessment (triaging) using an online assessment tool followed by a clinical interview via phone: a randomised controlled trial

**DOI:** 10.1186/s12888-025-07023-8

**Published:** 2025-07-01

**Authors:** Irosh Fernando, Rahul Gupta, Kate Simpson, Stuart Szwec, Mariko Carey, Agatha Conrad, Todd Heard, Lisa Lampe

**Affiliations:** 1 Hunter New England Local Health District, Newcastle, NSW Australia; 2 School of Medicine and Public Health, Faculty of Health and Medicine, University of Newcastle, Callaghan, NSW Australia; 3 Mental Health Line (MHL) and Northern Mental Health Emergency Care-Rural Access Program (NMHEC-RAP), Hunter New England Local Health District, Newcastle, NSW Australia; 4 Hunter Medical Research Institute, New Lambton, NSW Australia; 5 School of Medicine and Public Health, College of Health, Medicine and Wellbeing, University of Newcastle, Callaghan, NSW Australia; 6 Mental Health -Research, Evaluation, Analysis & Dissemination (MH-READ) Unit, Hunter New England Mental Health, Newcastle, NSW Australia; 7 School of Medicine and Public Health, University of Newcastle, Callaghan, NSW Australia; 8 Youth Justice, NSW Department of Communities and Justice, Newcastle, NSW Australia

**Keywords:** Online mental health assessment, Online psychiatry assessment, Online mental health triaging, Online questionnaire for mental health assessment, Randomised controlled trial, Web-based mental health assessment, Mental health assessment by self-reporting clinical information, Internet-based mental health assessment or triaging

## Abstract

**Background:**

The need for time-efficient and accessible mental health assessment is a priority in the face of high demand, limited resources and a progressive increase in the percentage of adults experiencing high or very high levels of psychological distress. Although there is broader supportive evidence for using online assessment as a potential solution, there is relatively little evidence from randomised controlled trials.

**Objective:**

To investigate whether patient online self-reported clinical information can save clinician time in subsequent mental health assessment via phone.

**Methods:**

Patients referred to a public mental health service by general practitioners via fax during business hours between February 2020 and June 2022 were randomly allocated to either the intervention (self-reporting of clinical information followed by clinician assessment by phone) or control (clinician phone assessment as usual) arm. The time to complete the assessment (call duration) was the primary outcome measure.

**Results:**

Out of 758 referrals assessed for eligibility, 377 (49.34%) entered the study and were randomised. Out of 184 referrals allocated to the intervention arm, the assessment was completed in 125, but only 81 were included in the analysis, mostly due to failure of clinicians to follow the protocol (completing the assessment without using self-reported data, likely due to inexperience with the novel process). Of 193 referrals allocated to the control arm, 135 completed the assessment and were included in the analysis. Average assessment completion time in the control arm was 25.19 min (standard deviation (SD) of 11.5 min) and 20.76 min (SD 7.49 min) in the intervention arm respectively, with a mean difference of 4.43 min (17.59% time reduction). When a mixed effects linear model was used to adjust for potential seasonal effect and correlation of outcome within clinicians, a statistically significant reduction of 3.29 min (*P* = 0.016, 95% CI (5.85, 0.73)) was still demonstrated by using online assessment.

**Conclusion:**

The use of online self-report clinical assessment by patients can save time to complete subsequent clinician assessment. Greater time-saving can be expected with better integration of this tool in workflow and increased clinician familiarity with using online self-reported data.

**Trial registration:**

(registered retrospectively) Registry: Australian and New Zealand Clinical Trial Registry (ACTRN). Registration number: anzctr.org.au ACTRN12624001293550. Date of registration: 24/10/2024.

**Supplementary Information:**

The online version contains supplementary material available at 10.1186/s12888-025-07023-8.

## Introduction

Patients seeking mental health care are often offered an initial assessment, which is known as triaging in many services across the world, particularly in emergency settings. Even though it is considered to be a relatively brief assessment, it needs to be comprehensive enough to support decision-making in relation to the urgency of care, managing risks, and arranging further assessments and necessary referrals; thus, it requires a skilled clinician to gather information including risks, individual needs and psychopathology [1].

According to the Global Burden of Diseases, Injuries, and Risk Factors Study (GBD) 2019, mental disorders remained among the top ten leading causes of burden worldwide; the global number of disability-adjusted life-years (DALYS) due to mental disorders increased from 80.8 million to 125.3 million between 1990 and 2019, thus requiring more effective services [2]. In NSW, Australia where this study was conducted, the percentage of adults experiencing high or very high levels of psychological distress measured by the Kessler 10 Plus (K10), increased from 9.8 to 17.7% between 2013 and 2019; in 2023 it was sitting at 18% overall but was much higher in Aboriginal adults (37%) according to the latest information available [3]. This trend is reflected nationwide, by an average annual rate of increase of 2.8% in mental health presentations to EDs between 2016 and 17 and 2020-21 [4].

The study was motivated by the need for time-efficient initial assessment (mental health triaging) in the face of high and increasing demand and limited resources. Mental health (MH) presentations to EDs have also become more acute with increased average annual rates of 13.7%, 9.4% and 4.7% respectively for triage categories of resuscitation, emergency, and urgent between 2016 and 17 and 2020-21, and more than a third not meeting required assessment timeframes [4] posing a risk of adverse outcomes and patient and carer dissatisfaction due to prolonged waiting time for assessment. As a novel solution to this immediate need, we developed an internet-based online assessment tool which guides patients and carers to supply pertinent information, including symptomatology and important cultural considerations.

The benefits of patient-entered data in computer-based interviews on a range of outcome measures including time-efficiency, feasibility, acceptability, and engagement of patients have been emphasised [5]. The feasibility of using a computerised self-assessment and its acceptability to patients has been demonstrated in outpatient clinics [6]; patients report finding the use of electronic devices to gather clinical information acceptable [7, 8]. Patients may even engage better with electronic assessment: it has been reported that patients preferred to disclose sensitive information, such as intimate partner violence, to a computer [9]; it has particularly been the case with young people [10] as they can disclose sensitive information without fear of judgment by health professionals [11]. Furthermore, patients have reported finding it helpful to organise their thoughts and communicate better, and that it improves the quality of their care [7]. However, none of these studies were randomised controlled trials, which has led to criticism of the research base [12]. In a recently published randomised controlled trial by the current team, the online self-report of clinical information through the use of a touchscreen mobile device prior to clinician assessment reduced clinical assessment time by nearly 10 min in an emergency department setting and was acceptable to the majority of patients and clinicians [13].

The current study seeks to add to the evidence base deriving from randomised controlled trials by extending investigation of online self-report of clinical information to an outpatient clinical setting. The study aimed to test whether initial online mental health self-report by community-based patients requiring a mental health assessment contributed to a reduction in the time required to complete the subsequent clinician assessment and whether this novel approach was acceptable.

### Design and development of the online assessment tool

Using the NSW Health mental health triaging template as a guide, clinical questions and the responses available for selection were designed by a team consisting of senior clinicians, including a panel of three psychiatrists and a clinical coordinator, in liaison with Aboriginal clinicians, and carer and consumer representatives. Online content was iteratively improved using feedback from Aboriginal clinicians, and carer and consumer representatives to make sure the content was culturally sensitive and easy to understand. A copy of the online assessment form is provided as a supplementary document.

## Method (Study design and setting)

The study was conducted at the Mental Health Line (MHL) located in the Hunter New England Local Health District (HNELHD). The MHL is a statewide phone service available via NSW Health 24 h a day, seven days a week offering brief assessment (triage), mental health advice, and recommendations [14]. If the mental health professionals of the MHL are concerned about the immediate safety of patients, they may advise the patients to present to their nearest ED.

This study was a randomised controlled trial (RCT) in which eligible and consenting clients referred by their GPs were randomised between the intervention arm ( mental health assessment using the online assessment tool prior to clinician assessment) and the control arm (existing assessment process) with an allocation ratio of 1:1. The randomisation sequence for patients was generated using SAS by the Data Sciences unit at the Hunter Medical Research Institute, and the sequence was kept concealed from both clinicians and patients. Patients in both the intervention and control arms experienced equivalent assessments as per the NSW Health Mental Health Triage Policy [15]. The study also assessed the acceptability of the assessment by inviting clients, carers, and clinicians to complete a satisfaction survey.

### Client and carer eligibility

English-speaking clients who were over 18 years of age and referred by their GPs, having basic computer literacy required for online assessment and having online access, were included in the study. Clients meeting any of the following conditions were excluded: under 18 years of age; unable to give consent due to intellectual disability, medical status or being acutely psychotic, manic or intoxicated. Clients in acute psychological distress and those requiring an interpreter, urgent psychiatric assessment as stated in the referral letter, and at increased risk of violence or suicide, were also excluded from the study.

### Recruitment of clients and carers and randomisation

**Fig. 1:**
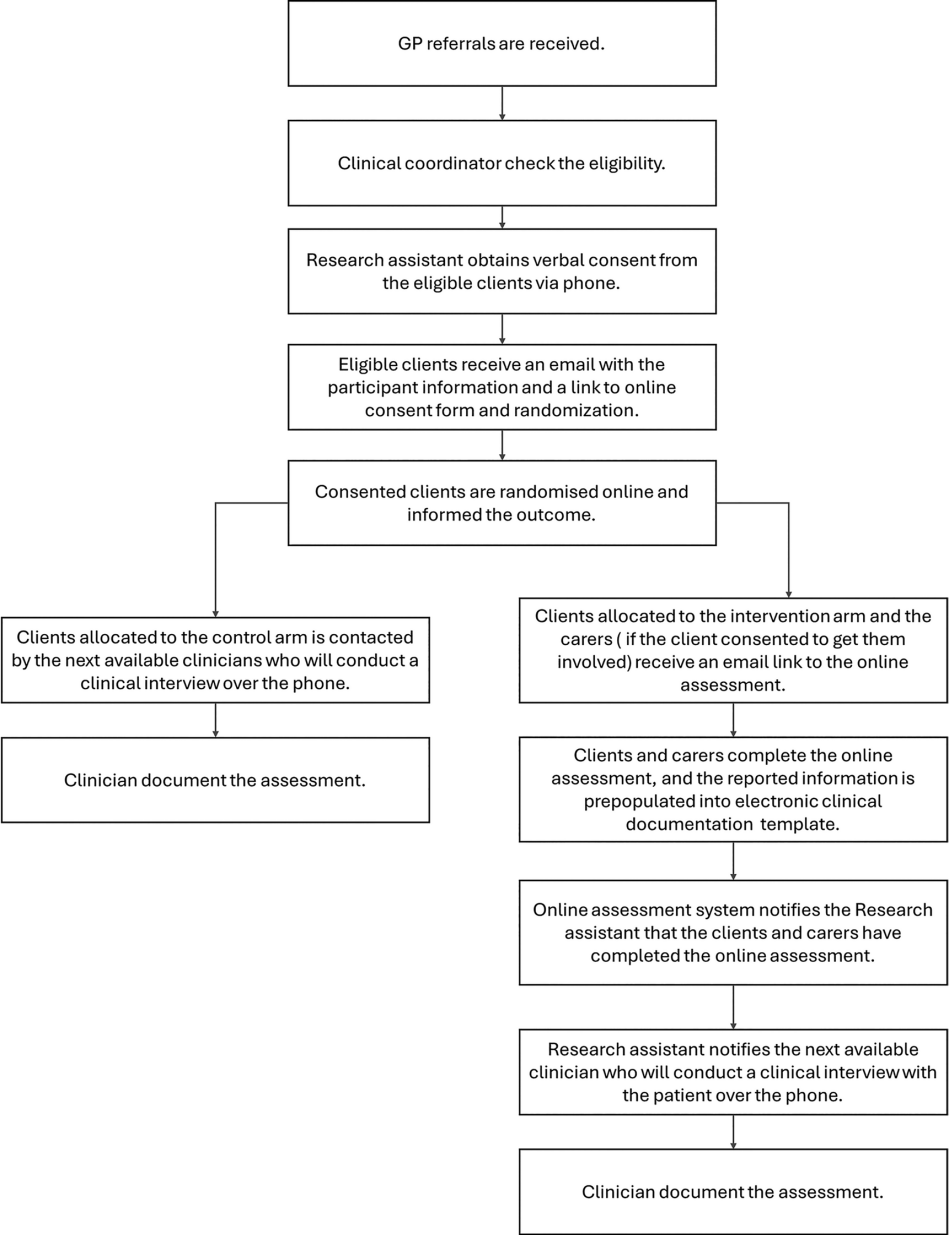
Flow diagram of the participants through the intake and assessment process

While the usual source of referrals for assessment at the study site includes direct phone calls from clients and referrals from GPs, other health professionals and third parties, only referrals from GPs were included in this study for reasons of feasibility in randomising the participants. Potential clients were identified and their eligibility for the trial determined by a senior clinician using the information provided in the referral. Clients who were deemed eligible were immediately contacted by the research assistant to confirm their eligibility and ascertain the potential involvement of a carer in the triaging, seeking permission from both parties for involvement in the assessment and the study. Once eligibility was confirmed and carer involvement determined, clients and participating carers were emailed further information about the study and a link for consenting online. Once consent was obtained, they were randomised by the online software program and informed if they were selected for online assessment or assessment as usual. The flow of the participants through the intake and assessment process is described in Fig. 1.

### Recruitment of triage clinicians and training to use the online triaging tool

All clinicians employed by the MHL were invited and deemed eligible to participate in the study. Information about the online assessment tool and consent form were provided and the consent was obtained by the research assistant. A training session including a demonstration of the use of the online assessment tool and training material was provided to the consenting triage clinicians.

### Experimental arm

Clients who were randomised to initial online assessment were sent a link for the online questionnaire, which comprised mostly checklists and also free text areas for written responses as required. Questions related to symptom screening, medical history, drug and alcohol use, current treatment, current functioning, carer responsibilities, current support, current accommodation, legal issues, previous mental health problems, family history, risk of self-harm or suicide, and risk of harm to others.

Once the client and any participating carers completed the online questionnaire, it prepopulated the clinical documentation template with the collected data and notified the research assistant. Then the next available clinician was allocated to the client. The protocol required the clinician to review the documentation and conduct an interview with the client over the phone. The interview was expected to be more focused and shorter given the client and carer had already self-reported most of the information that the clinician needed to gather. Clinicians were expected to use their own clinical judgement regarding the accuracy of the self-reported information and to verify information as clinically indicated. The information provided by clients and carers was presented separately and was not modifiable by the clinician, however, the clinician could add clinical notes prior to finalising the NSW Health assessment template clinical documentation. A sample copy of the assessment document is included as supplementary material.

If the participating client became distressed during completion of the online assessment, wished to withdraw from the study, or wanted to talk to a clinician, they were given the option to do so at any time. In that case, the client was directly contacted by the clinician and withdrawn from the study.

### Control arm

Clients in the control arm (i.e., assessment as usual) were directly contacted by clinicians who then conducted a clinical interview over the phone, collecting the clinical information required by the NSW Health mental health triaging template (including the same topics that clients in the intervention arm were asked online) and documenting it under relevant sections of the assessment template.

### Outcome measures

The main outcome measure was time to complete assessment via the phone interview all patients received as indicated by call duration, which was derived from routinely collected data by the health service. The interview time was expected to be shorter in the online assessment process because of the already available prepopulated client information which would otherwise all need to be gathered by the clinician. Similarly, the time for completing the clinical documentation was expected to be shorter. The secondary outcome measure was acceptability of online mental health assessment to clinicians and patients.

### Satisfaction survey

Clinicians were asked to rate their experience with online assessment on a five-point scale (1 very good; 5 very poor). Clients in the experimental arm and control arm were asked to rate their overall experience with their assessment on the same five-point scale.

## Statistical analysis and power analysis

Descriptive statistics were calculated for assessment call duration, clinician attributes, and the satisfaction survey. Continuous variable data is presented as mean (standard deviation; SD) or median (Q1, Q3), along with (min, max) depending on distribution. Responses to the satisfaction survey are presented as counts (%). Comparisons between survey responses (the proportions of ratings on the five-point scale) for patients allocated to the intervention arm and those allocated to control arm were performed using a Chi-squared test.

A mixed effects linear regression model was used to assess the effect of the online assessment on duration of assessment phone call. Fixed effects included allocation (control or intervention). Clinician experience in years with the health service and triaging were included as additional fixed effects but not interpreted in the results. A random effect for clinician was included to adjust for correlation of outcomes within clinicians providing triage. An additional random effect of study week was included to account for any week-to-week variability during the course of the study. Results presented show the difference in marginal means for call duration between control and intervention alongside a p-value. All statistical analyses were programmed using SAS v9.4 (SAS Institute Inc. Cary, NC, USA). Significance was set as α = 0.05 a priori.

**Sample size**: A sample of 160 clients randomised 1:1 (i.e., 80 per arm) was to give the study 80% power to detect a moderate effect size of 0.5 standard deviations (SD) at 5% significance. This calculation was inflated by 20% to account for potentially non-Normally distributed triage times. Historically, the duration of the assessment process ranges from approximately 30 to 90 min, with a mean of 60 min; this corresponds to a SD of approximately 15 min. The study was powered to detect a difference of approximately 8 min.

## Results

The study was conducted between February 2020 and June 2022 for two weekdays each week during 8.30am-4.30pm, based on the availability of research funding to employ the research assistant. The study faced delays, interruptions, and low recruitment during the Covid pandemic and ended after achieving the target recruitment.

**Fig. 2:**
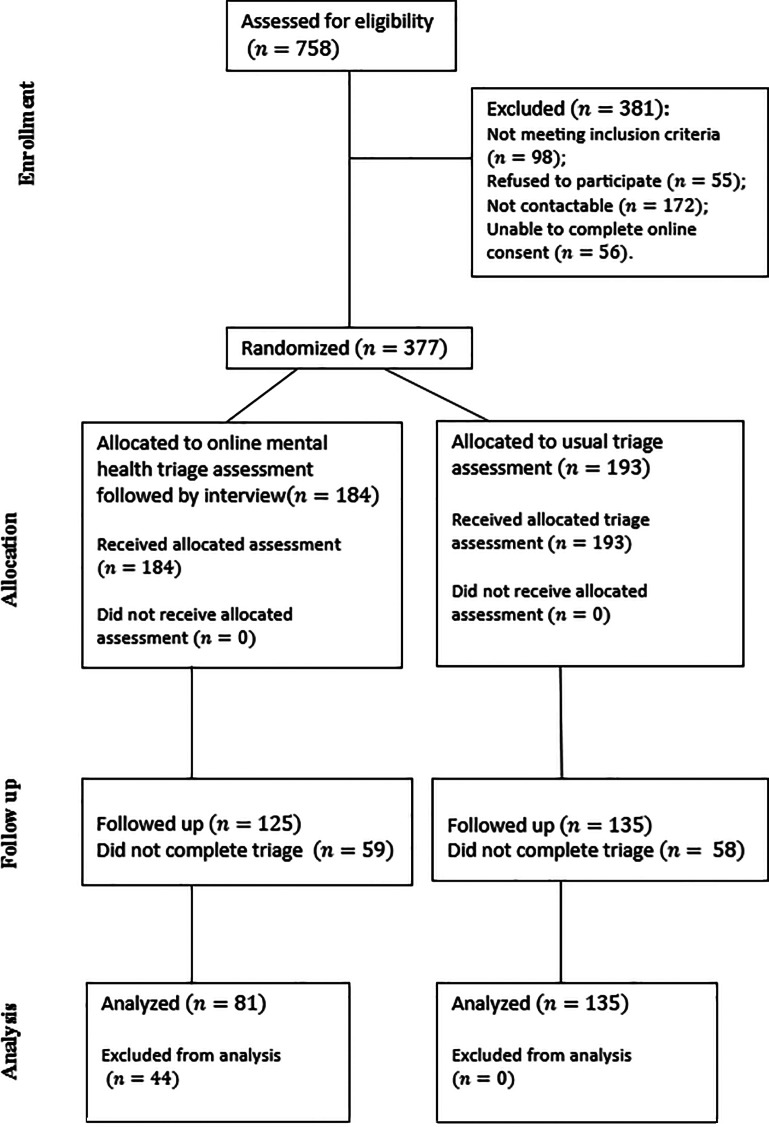
The flow of participants through each stage of the study

During the study period, a total of 758 referrals were assessed for eligibility: 172 (22.69%) were not contactable; 98 (12.93%) did not meet inclusion criteria; 55 (7.26%) declined consent; 56 (7.39%) were unable to complete the online consent process. Thus, 381 (50.26%) were excluded and 377 (49.34%) entered the study and were randomised. Out of 184 who were allocated to the intervention arm, 125 completed assessment. Only 81 were included in the analyses, with the remainder most commonly excluded due to clinician non-adherence to the protocol (mainly failure to use the system-generated clinical document prepopulated with self-reported patient and carer data, instead utilising a blank document, as occurs in the usual assessment process). Out of 193 clients who were allocated to the control arm, 135 completed assessment and were included in the analysis. Notably, the rates of non-completion of the assessment process were almost the same in each arm. There were no adverse or harmful events reported during the study. The flow of participants through each stage of the study is presented in a CONSORT [16] diagram ( Fig. 2).

A total of 37 clinicians participated in the study with their years of experience ranging between 6 months to 13 years (mean and standard deviation of 4.60 and 3.55 years respectively). Notably, 28 of the clinicians (76%) only used the online assessment two or fewer times during the study.

As shown in Table 1, average assessment call durations were 25.19 min (SD = 11.21) in the control arm and 20.76 min (SD = 7.43) in the intervention arm respectively, with an average reduction in call duration of 4.43 min (17.59% time reduction) in the intervention arm compared to the control arm average call time. Minimum call durations were comparable in both arms (4.28 min vs. 4.55 min in control and intervention arms respectively) whereas the maximum call duration in the control arm was 38 min higher than the maximum of the intervention arm (75.3 min vs. 37.4 min). Table 1. Summary of assessment duration (in minutes) between control and experimental arm.

**Table 1:** Comparision of call duration in the control and the intervention arms

		Arms		Total
		Control	Intervention	
Number of clients		135	81	216
Assessment call duration in minutes	Mean (SD)	25.19 (11.21)	20.76 (7.44)	23.53 (10.17)
	Median (Q1,Q3)	23.83 (17.65, 30.32)	19.83 (15.40, 25.52)	21.74 (16.50, 28.65)
	Minimum, maximum	4.28, 75.30	4.55, 37.35	4.28, 75.30

Results from the linear mixed effects model indicated that triage calls in the intervention arm had a statistically significant shorter duration of 3.29 min compared to the control arm (*P* = 0.016; 95% CI (0.73, 5.85)). The patient allocated to the control group with a call time of 75.3 min occurred due to completion of the original triage process, but the patient then disclosed another disorder that required further triage. Removal of this outlier results in a statistically significant shorter duration of 2.87 min (*P* = 0.011, 95% CI (0.66, 5.07).

Clinicians followed the patient allocation, so should have seen patients in both control and intervention arms. However, since the study was conducted only in business hours (due to funding limitations) and the service operates 24 h a day, despite a total of 37 clinicians participating in the study, not everyone had enough opportunity to use the online intervention to become familiar with it, and 14% of clinicians did not get the opportunity to use it at all. Thus, only 10 clinicians were able to complete the satisfaction survey. Of these 10 clinicians, 1 (10%) rated the online assessment as very good; 3 (30%) as good, 4 (40%) as neutral; 2 (20%) as poor; no clinicians rated it as very poor. In the control arm, 36 clients completed the satisfaction survey whereas only 26 clients in the intervention arm completed it. Carers were not included in the satisfaction survey. Most clients in both arms were satisfied with their assessment; the results from chi-square testing did not reveal statistically significant differences in ratings between experimental and control arms (*P* = 0.662). The results are summarised in Table 1.

**Table 2:** Summary clients’ rating on their overall experience with mental health assessment

Question	Response	Experimental arm (*n* = 26)	Control arm (*n* = 36)	Total	*P* value
How would you rate your overall experience with your mental health assessment?	Very good	11(42%)	17(47%)	28(45%)	0.662
	Good	9(35%)	9(25%)	18(29%)	
	Neutral	4(15%)	8(22%)	12(19%)	
	Poor	1(3.8%)	0(0%)	1(1.6%)	
	Very poor	1(3.8%)	2(5.6%)	3(4.8%)	

## Discussion

This paper makes an important contribution via a randomised controlled trial to the evidence base for efficient online mental health assessments which are feasible to implement and acceptable to clinicians and patients. While we were not able to demonstrate as great a time saving as expected the average saving of 3.29 min per assessment amounts to a 13% reduction in assessment time. In a 24-hour busy service completing 960 assessments per month on average, even the limited time savings demonstrated in the current study can make a meaningful difference. In our view, adoption of online self-report by a service as routine practice, supported by senior leadership and staff training offers the potential for greater time savings.

The finding of a higher maximum call duration in the control arm, where no patient-report data was available, could indicate that online self-report prior to clinician assessment might be particularly effective where patients present with high complexity. Clinical observation suggests that complex assessments need more time as more clinical information must be gathered, whereas assessments of well-known clients to the service, where much clinical information is already available in the medical record, results in shorter call durations. This possibility could be explored in future trials by including measures indicative of problem complexity.

We acknowledge several limitations of this study. Firstly, the study did not assess the influence of clinician and patient characteristics on the outcome. We did not assess clinician attitudes towards online assessment, as a means of determining possible bias. Also, the study did not assess the client time for completing the online assessment. Future studies will benefit from controlling for characteristics such as clinician experience level and patient complexity. Secondly, the study was not representative of the entire population of mental health assessment since it excluded self-referrals by clients and third parties, and acutely distressed or suicidal client. It is thus reasonable to assume that the study sample may have been less acute compared to the excluded population; however, it is of note that a recent study by the current authors in an emergency department setting confirmed time savings and acceptability in a more acute population [13]. Thirdly, clinician inexperience in using online assessment is a significant limitation of the study, which likely unfavourably affected the outcome and also resulted in 44 out of 125 participants who completed the intervention being excluded from the analysis due to clinician non-adherence to the study protocol. An intention to treat analysis including the excluded participants would have produced more accurate results but we were unable to do so since the call duration of the excluded participants had not been flagged and therefore could not be retrieved. As noted, 35% of the clinicians used the online assessment only once, and altogether 62% of the clinicians only used it once or twice; an additional 14% never used the online assessment system. Thus, future research should focus on improved training and perhaps requiring demonstration of clinician proficiency as an inclusion criterion. It is expected that further time saving would be realised if clinicians had more experience in using online assessment, which might allow them to use it more efficiently.

It is also important to note that the online assessment tool was developed at minimum cost, with limited resources and under significant time constraints by active and busy clinicians to support clinically focussed research, without the funding or aim of making it a commercial clinical application. There is great potential to improve the user-interface of the online assessment and also make the content of the assessment more concise so that it can be used more efficiently.

As it was not possible to blind participants to conditions, clinician bias towards either the intervention or control could not be excluded. A negative bias towards the online instrument could adversely influence how effectively the online assessment was used and the outcome measures. Clinicians may also have been hesitant about using a new process with which they were not familiar. In future studies it may be helpful to use qualitative methodology to explore the clinician, carer and patient barriers to using online assessment.

The increasing demand for mental health assessments and resource constraints reflects a global situation. The solution we have developed may have application globally in settings where internet access is readily available. Through the scaffolding of a comprehensive initial psychiatric assessment, it may also support clinicians who are less experienced in mental health assessment. Importantly, it can save time in any subsequent specialist assessment, presenting the opportunity for high-quality stepped care in contexts where specialist clinicians are a scarce resource. Overall, this paper presents evidence for an efficient service delivery model for patients seeking mental health assessment, potentially accessible to any patient with a mobile device.

## Electronic supplementary material

Below is the link to the electronic supplementary material.

Supplementary Material 1

Supplementary Material 2

Supplementary Material 3

Supplementary Material 4

## Data Availability

The datasets generated and/or analysed during the current study are available in the ANZCTR repository, https://www.anzctr.org.au/Trial/Registration/TrialReview.aspx?ACTRN=12624001293550.
