# Supplementary material for: Improving the time-efficiency of initial mental health assessment (triaging) using an online assessment tool followed by a clinical interview via phone: a randomised controlled trial
Source: BMC Psychiatry. 2025 Jul 1;25:635. doi: 10.1186/s12888-025-07023-8 (PMC12220592; doi:10.1186/s12888-025-07023-8)
Supplement: Supplementary file 1 — Supplementary Material 1 [file 12888_2025_7023_MOESM1_ESM.pdf]

Name: Client 21_28-01 Client 21

DOB:

Contact No:

Carer Name:

Aboriginal or Torres Strait Islander Yes

Would like an Aboriginal health worker if available Yes

Alerts and Risks [ ]Yes [ ]No

Summary *(summarise after triage completed):*
--

TRIAGE DETAILS

Communication Issues
Communication Issues

Reason for Referral *Include whether client is opposed to referral*
Who recommended you to this health service? GP,
Reason for Referral:
Client agreed with the Referral

Comments by Clinician
--

HISTORY *Presenting problems reported by client*
DEPRESSION SYMPTOMS
Suicidal thoughts
ANXIETY
Panic attacks
Feeling nervous, anxious or on edge
PSYCHOTIC SYMPTOMS
Seeing things or hearing things that were not there (hallucinatory experiences)
Feeling paranoid or suspicious about people
OTHER_SYMPTOMS:

Other Concerns *Reported by carer*
--

Past Psychiatric History *Reported by client*
Previous MH history: Had mental health problems in past
Previous symptoms were: depressed

Past MH diagnosis: diagnosed with a mental illness
History of Trauma/abuse:
Experienced previous trauma
Experienced Physical Trauma, Experienced Sexual Trauma , Experienced Emotional Trauma.
In childhood
Family MH history: YES Depression
Family member suicide: NO

Comments by Clinician
--

GP Contacts
--

MEDICAL ISSUES *significant illnesses,allergies,adverse drug reactionsdelrium risk, pregnancy*
--
Comments by Clinician
--

CURRENT TREATMENTS *e.g. medications psychological interventions, complementary/alternative interventions, providerss/services involved/*
Reported by Client
Currently using prescribed medication
zoloft 100mg
Taking Medication sometimes
Experiences side effects
Receiving Therapy or counselling
Recieving alternative treatment

Reported by Carer
--
Comments by Clinician
- -

DRUG AND ALCOHOL USE
Reported by Client
Have used too much alcohol
Craving for alcohol
Illegal drug use:
Cannabis,
Did you use these drugs more than you intended to or did their use cause any problems: YES

Reported by Carer
--
Comments by Clinician
- -

CURRENT FUNCTIONING AND SUPPORTS *e.g. concerns regarding living situation, parental or other responsibilities, educational difficulties (C&A), social functioning, note name, age,*

current whereabouts of dependent/s:
Reported by Client
Employment Status: Unemplyed
Not performing usual activities
- Difficulty with activities of daily living
- Difficulty with home duties
- Difficulty with care of dependents
Carer Responsibilities: YES
Children
Parents
If you are a carer for your children, are you concerned about their well-being? YES
Accommodation: Hostel
No concerns about accomodation

Reported by Carer
--
Comments by Clinician
- -

LEGAL STATUS/ FORENSIC ISSUES *e.g guardianship, community treatment orders, recent incarceration, police matters*

Reported by Client
Charges, convictions, or custodial sentences? No
Family law case? No
Involved in civil case or action? No
Workers compensation or insurance matter? No
previous charges, convictions, custodial sentences? No
Do you have a guardianship order? No
currently on a mental health order? No

Comments by Clinician
--

MENTAL STATE IMPRESSION *e.g consider information provided by client and other sources*

eg. sleep, Eating, Speech, Mood, Thoughts Perception, Cognition, Insight and Judgement
--
Comments by Clinician
--

POSSIBLE RISKS

Suicide

Significant past history of risk	[ ]Yes	[ ]No	[ ]Unknown
Recent thoughts,plans,symptoms indicating risk	[ ]Yes	[ ]No	[ ]Unknown
Recent behaviour suggesting risk	[ ]Yes	[ ]No	[ ]Unknown

Concern from others about risk<br>*(assessment should include corroboration where possible)*	[ ]Yes [ ]No [ ]Unknown

Risks Reported by Client
I have thought about how I would commit suicide
I feel at risk that I might act on these thoughts and attempt suicide
I have attempted suicide
I have attempted suicide more than once
I intended to end my life
I was admitted to a psychiatric ward
I have been recently experiencing thoughts of self-harm without intending suicide: Several times a week

Risks Reported by Carer
--

Risks Comments by Clinician
- -

Violence

Significant past history of risk	[ ]Yes [ ]No [ ]Unknown
Recent thoughts,plans,symptoms indicating risk	[ ]Yes [ ]No [ ]Unknown
Recent behaviour suggesting risk	[ ]Yes [ ]No [ ]Unknown
Concern from others about risk<br>*(assessment should include corroboration where possible)*	[ ]Yes [ ]No [ ]Unknown

Risks Reported by Client
I have been recently experiencing any thoughts of harming others
I have been been violent or aggressive towards others
The following risks are affecting me
I experience domestic violence
I am concerned about the safety of my home situation
I am feeling isolated
I do not have good access to help or health services

Risks Reported by Carer
--

Risks Comments by Clinician
- -

**Current problems with alcohol or substance misuse**	[ ]Yes [ ]No [ ]Unknown
**Major mental illness or disorder**	[ ]Yes [ ]No [ ]Unknown
**At risk mental state**<br>*(e.g. depressed, hopelessness, despair, guilt, marked agitation, disorganisation, intoxication etc).*	[ ]Yes [ ]No [ ]Unknown
**Person's level of risk appears to be highly changeable**<br>*(assessment should include corroboration where possible)*	[ ]Yes [ ]No [ ]Unknown
**Significant uncertainty in the assessment of the level of risk**<br>*(assessment should include corroboration where possible)*	[ ]Yes [ ]No

Overall Level of Risk

Suicide	[ ]Yes [ ]No [ ]Unknown
Violence	[ ]Yes [ ]No [ ]Unknown
**Other*** specify___________________________	[ ]Yes [ ]No [ ]Unknown
**Other*** specify___________________________	[ ]Yes [ ]No [ ]Unknown

**Consider other risks e.g. self-harm, child safety, absconding, exploitation, domestic violence, abuse, neglect, environment risks.*

Urgency of Response
PLAN

SUMMARY

Overall clinical impression, including possible risks and history of impulsivity

CRISIS TRIAGE RATING SCALE

RATING A: Dangerousness

[ ] 1 Expresses or hallucinates (hears commands) suicidal/homicidal ideas or has made a serious attempt in present episode of illness. Unpredictable, impulsive, violent.

[ ] 2 Expresses or hallucinates suicidal/homicidal ideas, without conviction, or the behaviour is somewhat dependent on the stress in the environment. History of violence or impulsive behaviour, but no current signs of this.
[ ] 3 Some suicidal/homicidal ideas with ambivalence, or made only ineffectual gestures. Questionable impulse control.
[ ] 4 Some suicidal/homicidal ideation or behaviour. or history of same, but clearly wishes and is able to control behaviour.
[ ] 5 No suicidal/homicidal ideation/behaviour. No history of violence or impulsive behaviour.

RATING B: Support system
[ ] 1 No family, friends or others. Agencies cannot provide the immediate support needed.
[ ] 2 Some support can be mobilised, but its effectiveness will be limited.
[ ] 3 Support system potentially available, but significant difficulties exist in mobilising it.
[ ] 4 Interested family, friends or others, but some question exists of ability or willingness to provide support needed.
[ ] 5 Interested family, friends or others able and willing to provide support needed.

RATING C: Ability to cooperate
[ ] 1 Unable to cooperate or actively refuses.
[ ] 2 Shows little interest or comprehension of efforts made on their behalf.
[ ] 3 Passively accepts intervention strategies.
[ ] 4 Wants help but is ambivalent or motivation is not strong
[ ] 5 Actively seeks treatment, willing and able to cooperate

RATING SCALE (CTRS)
[ ] Rating A: Dangerousness
[ ] Rating B: Support System
[ ] Rating C: Ability to Cooperate
[ ] **CTRS total score (A+B+C)**
This scale is to be used in conjunction with the triage information to make an informed decision about the urgency of response.

ACTION PLAN
Urgency of Response Scale

[ ] **Category A: 3-9 total score**
- Extreme Urgency: Immediate response requiring Police/Ambulance or other service (e.g., overdose, siege, imminent violence)
[ ]Police notified:
[ ] Ambulance notified:
[ ] Inpatient Mental Health service notified:
[ ] Emergency Department notified:
[ ] Other:

Details of Action Plan:

[ ] **Category B: 10 total score**
- High Urgency: see within 2 hours/present to Psychiatric Emergency Service or Emergency

Department in General Hospital (e.g., acute suicidality, threatening violence, acute severe non-recurrent stress)
[ ] Inpatient Mental Health service notified:
[ ] Emergency Department notified:
[ ] Other:
Details of Action Plan:

[ ] **Category C: 11 total score**
 - **Medium Urgency:** see within 12 hours (e.g. moderate distress, suicidal ideation of moderate to severe nature, disturbed behaviour)
[ ] Referred to Community Mental Health service:
[ ] Referred to Community Health:
[ ] Other:
Details of Action Plan:

 - **Category D 12-13 total score**
 - **Low urgency:** see within 48 hours (e.g. moderate distress, has some supports in place but situation becoming more tenuous)
[ ]Referred to Community Mental Health service:
[ ]Referred to Community Health:
[ ] Other:
Details of Action Plan:

[ ] **Category E 14-15: total score**
 - **Non Urgent:** see within 2 weeks
[ ] Referred to Community Mental Health service:
[ ] Referred to Community Health:
[ ] Referred to GP:
[ ] Other:
Details of Action Plan:

[ ] **Category F** - Requires further triage contact/follow up
[ ] Referred to Community Mental Health service:
[ ] Referred to Community Health:
[ ] Referred to GP:
[ ] Other:
Details of Action Plan:

[ ] **Category G** - No further action required

Complete ALERTS/RISKS and Summary at start of form
