# Supplementary material for: Improving the time-efficiency of initial mental health assessment (triaging) using an online assessment tool followed by a clinical interview via phone: a randomised controlled trial
Source: BMC Psychiatry. 2025 Jul 1;25:635. doi: 10.1186/s12888-025-07023-8 (PMC12220592; doi:10.1186/s12888-025-07023-8)
Supplement: Supplementary file 3 — Supplementary Material 3 [file 12888_2025_7023_MOESM3_ESM.pdf]

# HNE Mental Health Contact Centre

## TRIAGE - Carer

### Client Name Rahul test

**CARER First Name** ☐ **Surname** ☐

## Current support

**What is your relationship to the referred person (choose all that apply)?**

☐ Designated carer
☐ Principal Care Provider
☐ Relative
☐ Friend
☐ Other

**Do you require further help in supporting the person you are caring for?** ○ Yes ○ No

**How often are you in contact with this person?** ○ At least daily ○ Several times per week ○ About weekly ○ 1-2 times per month ○ Other

**Current functioning and risk of harm**

Please select all your concerns regarding the person from the following list:

☐ Difficulties in caring for themselves or function independently
☐ Difficulties in managing their finances
☐ Difficulties with travel
☐ Difficulties with independent tasks ( e.g. cooking, shopping, house cleaning, managing finances
☐ Difficulties with personal care (e.g. personal hygiene)
☐ The person is not able to control his/her bowel and bladder
☐ The person is at risk of exploitation e.g. financial, accommodation
☐ At risk of wandering away from home or getting lost
☐ At risk of falling
☐ Unable to use appliances safely in the home
☐ At risk of violence from others
☐ At risk of harm to their reputation because of their behaviour or symptoms

**Is the person currently experiencing any domestic violence or physical abuse?** ○ Yes ○ No ○ Unknown

**Does the person currently experience any sexual abuse?** ○ Yes ○ No ○ Unknown

**Is the person currently employed?** ○ Yes ○ No ○ Unknown
**If Yes: Is the person coping well at work?** ○ Yes ○ No ○ Unknown
**Are you concerned that their job is at risk due to their current symptoms or behaviour?** ○ Yes ○ No ○ Unknown

## Drug and alcohol use

**Please indicate whether you have concerns about any of the following:**
☐ Increase in alcohol use
☐ Use of illicit drugs
**If Yes**
**Please indicate whether the following are being used (select all that apply):**
☐ Cannabis
☐ Opiates
☐ Amphetamine/methamphetamine (including Ice)
☐ Ecstasy
☐ Other ( e.g. prescription medications)

## Risk of self-harm or suicide

**Are you concerned that person is at risk of suicide or self-harm?** ○ Yes ○ No ○ Unknown
**Does the person have a history of self-harm/injury?** ○ Yes ○ No ○ Unknown
**Does the person have a history of attempted suicide?** ○ Yes ○ No ○ Unknown

**Does the person have access to firearms?** ○ Yes ○ No ○ Unknown
**If yes, have you informed police?** ○ Yes ○ No

## Child well-being

**Are there children in the person's care?** ○ Yes ○ No ○ Unknown
**If Yes**
**Do you have any concerns about this (e.g. about the well-being of any children?)**
○ Yes ○ No
**Please indicate what your concerns are (select all that apply):**
☐ Physical abuse
☐ Emotional abuse
☐ Sexual abuse
☐ Neglect
☐ Any other, please give detail:

## Current treatment

**Is the person currently receiving treatment for a mental illness or condition?** ○ Yes ○ No ○ Unknown

**If Yes**

**Do you have any concerns about the adequacy of the person's treatment?** ○ Yes ○ No ○ Have not thought about it

**Where are they receiving treatment (select all that apply):**

☐ GP or family doctor
☐ Psychologist or counsellor
☐ Private psychiatrist
☐ Community mental health centre
☐ Any other, please give detail:

**Has medication been prescribed?** ○ Yes ○ No ○ Unknown

**If Yes**

**Does the person take the medication as prescribed?** ○ Yes ○ No ○ Do not Know

**Do you have an Awareness of Support Services for Carers?** ○ Yes ○ No

**If you have any concerns that have not been addressed in this questionnaire, please list them.**

SUBMIT
