# Supplementary material for: Improving the time-efficiency of initial mental health assessment (triaging) using an online assessment tool followed by a clinical interview via phone: a randomised controlled trial
Source: BMC Psychiatry. 2025 Jul 1;25:635. doi: 10.1186/s12888-025-07023-8 (PMC12220592; doi:10.1186/s12888-025-07023-8)
Supplement: Supplementary file 4 — Supplementary Material 4 [file 12888_2025_7023_MOESM4_ESM.pdf]

HNE Mental Health Line TRIAGE form

Welcome to the service. Please answer the following questions as far as you are able to. The information will assist us to provide the most appropriate healthcare to you.

Communication

Do you have cultural or language barriers in using the Mental Health Service?
○ Yes ○ No

If 'Yes': Please select which of the following you would like us to provide if possible:
Interpreter ☐ Language: [ ]
Aboriginal health liaison worker ☐
Other (please specify): [ ]

About

Please choose all the boxes that describe your background.
- ☐ Aboriginal
- ☐ Torres Strait Islander
- ☐ Immigrant
- ☐ Refugee
- ☐ Member of an ethnic minority group

What is your current gender identity?

(Check all that apply)
- ☐ Male
- ☐ Female
- ☐ Female-to-Male (FTM)/Transgender Male/ Trans Man
- ☐ Male-to-Female (MTF)/Transgender Female/Trans Woman
- ☐ Genderqueer, neither exclusively male nor female
- ☐ Additional Gender Category/(or Other)
- ☐ Prefer not to say

Employment and income

(Check all that apply)
- ☐ employed full time
- ☐ employed part time
- ☐ unemployed
- ☐ Disability Support pension
- ☐ Age pension

Carer responsibilities

Is someone dependent on you, or are you caring for someone? ○ Yes ○ No

If 'yes' Please select your carer responsibilities:
- ☐ Spouse/partner
- ☐ Children
- ☐ Parents

☐ Friend
☐ Relative
☐ other

If you are a carer for your children, are you concerned about their well-being? ○ Yes ○ No

Referral Details

Who referred you to this service?

☐ GP
☐ Myself
☐ Family/carer
☐ None of the above

Please describe the reason for the referral

Do you agree to the referral ○ Yes ○ No ○ Don't Know

If No please indicate why you do not think the referral is a good idea (select all that apply):

☐ I prefer to manage myself;
☐ I do not have a problem
☐ I do not feel that this service can help me;
☐ I do not think that this service offers type of treatment that I need
☐ Other

Mental Health Concerns

Please select the symptons you are experiencing now

Depression Symptons

Please select all that apply to you.

☐ Low mood all the time or most of the time
☐ Loss of interest in activities that you normally enjoy
☐ Self-harm thoughts
☐ Suicidal thoughts
☐ Lack of energy or feeling tired
☐ Difficulty in falling asleep and/or waking up intermittently
☐ Sleeping too much
☐ Loss of appetite
☐ Eating too much
☐ Lack of concentration
☐ Feeling guilt or blaming yourself excessively
☐ Feeling worthless
☐ Feeling hopeless

Anxiety Symptoms

☐ Worrying about being judged by other people
☐ Avoid social situations or interacting with people
☐ Worrying too much (e.g. worrying about anything and everything)
☐ Feeling restless and/or agitated
☐ Panic attacks (a sudden overwhelming feeling of acute and disabling anxiety)
☐ Feeling nervous, anxious or on edge

Do you experience the following symptons if you feel nervous
☐ Heart racing
☐ Tightness in your chest
☐ Tingling sensation
☐ Feeling dizzy
☐ Sweating
☐ Feel nauseous or sick in your stomach
☐ A nervous feeling in your stomach (e.g. feeling butterflies or a knot in your stomach)

Eating disorder symptoms

☐ Keeping your weight so low that others were worried about you
☐ Binge eating
☐ Doing excessive physical exercise in order to lose weight
☐ Skipping meals in order to lose weight
☐ Purging after meals

Other Symptoms

☐ Seeing things or hearing things that were not there (hallucinatory experiences)
☐ Feeling paranoid or suspicious about people
☐ Reduced need for sleep
☐ Unusually high energy
☐ Feeling unusually powerful or important
☐ Talking too fast or over the top of others
☐ Overspending
☐ Increased sex drive

Any other symptoms (please describe)

Current functioning

Are you currently able to perform all your usual activities about as well as you would like to? ○ Yes ○ No

If No What activities are you unable to perform or do you need assistance with? (Select all that apply)
☐ activities of daily living (e.g. preparing meals for yourself, getting dressed, showering or bathing)
☐ home duties you need to do, such as shopping, gardening or cleaning the house
☐ travel by yourself, either driving yourself or taking public transport

☐ Work duties (if you are employed in any capacity)
☐ Caring for those who are dependent on you (e.g. children, elderly, disabled)

Current support

Do you have a carer or support person? ○ Yes ○ No

If 'yes' please list carer/s and support person/s that you would like to be involved in your treatment.

Current accommodation

What type of place do you live in?

○ Rental Property (private or housing NSW)
○ Own house
○ Refuge
○ Hostel
○ Homeless

Do you have any concerns about your accommodation? ○ Yes ○ No

Legal issues

It can be helpful for us to be aware of any legal issues you may have. Please answer the following questions.

1) Do you have any current legal issues?
   a. charges, convictions, or custodial sentences? ○Yes ○No ○Prefer not to say
   b. Family law case ○Yes ○No ○Prefer not to say
   c. Involved in civil case or action ○Yes ○No ○Prefer not to say
   d. Workers compensation or insurance matter ○Yes ○No ○Prefer not to say

2) Do you have any previous charges, convictions, custodial sentences? ○Yes ○ No ○Prefer not to say

3) Do you have a guardianship order? ○Yes ○No ○Prefer not to say

4) Are you currently on a mental health order? ○Yes ○No ○Prefer not to say

Drug and alcohol use (Current)

1) Has there been a time where you used to drink too much alcohol? ○Yes ○No
If the answer is 'yes' to the previous question please select all the symptoms that you experienced during that time (either when drinking or between drinks):
☐ Craving for alcohol
☐ Nausea
☐ Tremors
☐ Sweats
☐ Feeling confused or disoriented
☐ Seeing or hearing things (hallucinatory experiences) that didn't exist

If 'yes' Please select all the treatments that you received :
☐ I didn't have any treatment
☐ Counselling
☐ Group therapy
☐ Medication
☐ Admission to a Drug and Alcohol Facility or rehab
☐ Other

Have you used any of the following illegal (recreational) drugs in the past (please select)
☐ Cannabis (marijuana)
☐ Amphetamine (speed)
☐ Heroin
☐ Ice (methamphetamine)
☐ Cocaine

Did you use ever use any of these drugs more than you intended to, or did their use cause any problems e.g. financial, work, relationship problems
○ Yes
○ No

Current Treatments

Have you been prescribed any medications to take? ○ Yes ○ No
If the answer is 'yes' to the previous question, please provide the following details for each medication (you can skip this if you have a referral from your GP which outlines your current medication)
Name of the medications and dose you are taking

Are you taking the prescribed medication regularly? ○ Yes ○ No ○ Sometimes
Do you have any troublesome side effects from it? ○ Yes ○ No
Are you receiving any therapy or counselling currently (except for drug and alcohol)? ○ Yes ○ No

Please select all the services or support that you receive:
☐ NDIS funding
☐ House with No Steps/ Life Without Barriers (or any other non-governmental organisations)

Do you receive any alternative, traditional or culturally relevant treatments? ○ Yes ○ No

Trauma history

Have you ever experienced any trauma? ○ Yes ○ No
Have you experienced any torture? ○ Yes ○ No

If the answer is 'yes' to either previous question, it would be helpful for us to know a little more, if you are able or willing to tell us the Nature of trauma:

☐ Physical
☐ Sexual
☐ Emotional

Please select when you experienced the trauma:
○ During my childhood
○ More than 10 years ago
○ About 5- 10 years ago
○ About 3-5 years ago
○ About 1-3 years ago
○ Within the last 12 months

Previous Mental Health Problems

Have you experienced mental health problems in the past? ○ Yes ○ No

If Yes please describe the sypmtoms

Have you ever been diagnosed with a mental illness? ○ Yes ○ No ○ Don't Know

Family history of mental illnesses

Has anyone in your family suffered any mental illness or dementia? ○ Yes ○ No ○ Don't Know

If 'yes' please select the diagnoses:
○ Depression
○ Anxiety
○ Dementia
○ psychosis/schizophrenia
○ Mania/bipolar disorder
○ Other condition
○ Don't Know

Has anyone in your family taken their own life by suicide? ○ Yes ○ No

Risk of self-harm or suicide

Have you been recently experiencing thoughts of suicide? ○ Yes ○ No

If 'yes' how often do you experience these thoughts:
○ Daily Daily
○ Several times a week Several times a week
○ Several times a month Several times a month

Have you thought about how you would commit suicide? ○ Yes ○ NO

If 'yes' do you feel at risk that you might act on these thoughts and attempt suicide? ○ Yes ○ NO

Have you attempted suicide ? ○ Yes ○ No

If the answer is ‘yes’ to the above question, please select all the relevant check boxes:

- ☐ I have attempted suicide more than once
- ☐ I intended to end my life
- ☐ I was treated at the Emergency Department
- ☐ I was admitted to a medical ward
- ☐ I was admitted to ICU
- ☐ I was assessed by Mental Health Team
- ☐ I was admitted to a psychiatric ward
- ☐ I was followed up by Community Mental Health Team

Have you been recently experiencing thoughts of self-harm without intending suicide?
○ Yes ○ No

If the answer is ‘yes’ to the above question, how often do you experience these thoughts:

- ○ Daily
- ○ Several times a week
- ○ Several times a month

Risk of harm to others

Have you been recently experiencing any thoughts of harming others? ○ Yes ○ NO

Have you ever been violent or aggressive towards others? ○ Yes ○ NO

Sexual identity conflicts

Do you feel uncomfortable with your sexual identity (e.g. feeling uncomfortable with your gender) ? ○ Yes ○ NO

Risks to the person

Please indicate whether any of the following risks are currently affecting you:

- ☐ I experience domestic violence
- ☐ I experience any physical abuse
- ☐ I experience sexual abuse
- ☐ People are taking advantage of me
- ☐ I am concerned that I may get confused, wander and get lost
- ☐ I am concerned that I may damage my reputation because of my illness
- ☐ I am unsteady on my feet or concerned that I may fall and injure myself
- ☐ I am concerned that I may lose my job/employment or become unemployed
- ☐ I am concerned about the safety of my home situation (e.g. fire, burglary)
- ☐ I am feeling isolated
- ☐ I do not have good access to help or health services

Access to firearms

Do you have access to firearms? ○ Yes ○ NO

[SAVE] [FINISHED]
